# *C. elegans *serine-threonine kinase KIN-29 modulates TGFβ signaling and regulates body size formation

**DOI:** 10.1186/1471-213X-5-8

**Published:** 2005-04-19

**Authors:** Lisa L Maduzia, Andrew F Roberts, Huang Wang, Xia Lin, Lena J Chin, Cole M Zimmerman, Stephen Cohen, Xin-Hua Feng, Richard W Padgett

**Affiliations:** 1 Waksman Institute, Department of Molecular Biology and Biochemistry, and Cancer Institute of New Jersey, Rutgers University, Piscataway, NJ 08854-8020, USA; 2 Michael E. DeBakey Department of Surgery, Baylor College of Medicine, Houston, TX 77030, USA; 3 Department of Molecular and Cellular Biology, Baylor College of Medicine, Houston, TX 77030, USA

## Abstract

**Background:**

In *C. elegans *there are two well-defined TGFβ-like signaling pathways. The Sma/Mab pathway affects body size morphogenesis, male tail development and spicule formation while the Daf pathway regulates entry into and exit out of the dauer state. To identify additional factors that modulate TGFβ signaling in the Sma/Mab pathway, we have undertaken a genetic screen for small animals and have identified *kin-29*.

**Results:**

*kin-29 *encodes a protein with a cytoplasmic serine-threonine kinase and a novel C-terminal domain. The kinase domain is a distantly related member of the EMK (ELKL motif kinase) family, which interacts with microtubules. We show that the serine-threonine kinase domain has *in vitro *activity. *kin-29 *mutations result in small animals, but do not affect male tail morphology as do several of the Sma/Mab signal transducers. Adult worms are smaller than the wild-type, but also develop more slowly. Rescue by *kin-29 *is achieved by expression in neurons or in the hypodermis. Interaction with the dauer pathway is observed in double mutant combinations, which have been seen with Sma/Mab pathway mutants. We show that *kin-29 *is epistatic to the ligand *dbl-1*, and lies upstream of the Sma/Mab pathway target gene, *lon-1*.

**Conclusion:**

*kin-29 *is a new modulator of the Sma/Mab pathway. It functions in neurons and in the hypodermis to regulate body size, but does not affect all TGFβ outputs, such as tail morphogenesis.

## Background

The transforming growth factor β (TGFβ) superfamily is involved in many developmental decisions from primitive animals such as *Cnidaria *and sponges to higher animals [1-4]. The core of the signaling pathway has been elucidated in the last few years and reveals a rather simple signaling cascade. These ligands transmit the TGFβ signal by binding transmembrane receptor serine-threonine kinases (RSKs). Once ligand is bound, the type II RSK phosphorylates the type I RSK in a cytoplasmic region rich in glycine and serine residues (GS domain). Phosphorylation activates the type I RSK and enables it to phosphorylate downstream mediators referred to as the Smads. Once the receptor-regulated Smads (R-Smads) are phosphorylated, they are able to physically interact with another subset of Smads identified as the common Smads (Co-Smads) and translocate to the nucleus where they affect target gene transcription [3,5-7].

Of the five TGFβ-like ligands in *C. elegans*, *dbl-1 *(*dpp *and BMP-like) and *daf-7 *(dauer formation abnormal) are the best characterized. *dbl-1 *transmits the Sma/Mab (small/male tail abnormal) pathway signal while *daf-7 *regulates formation of dauer, an alternative life stage entered in response to low food or high population density [8-10]. These two pathways share a common type II RSK, *daf-4*. *daf-4 *animals are small, exhibit fused male tail sensory rays and constitutively form dauer larvae [11,12]. Mutants of all other known components of the dauer pathway are either dauer constitutive (*daf-c*) like *daf-7 *or dauer defective (*daf-d*) [3,13]. Based on the Sma/Mab phenotypes of *daf-4 *mutants, *sma-2*, *sma-3*, *sma-4 *and *sma-6 *were identified and cloned. *sma-2*, *sma-3 *and *sma-4 *encode Smads while *sma-6 *encodes a type I RSK [12,14]. The Sma/Mab signal is transmitted upon binding of the ligand, DBL-1, to the type II and type I RSKs, DAF-4 and SMA-6 respectively. Once stimulated, SMA-6 activates the Smads, SMA-2, -3 and -4, causing them to affect target gene transcription.

Although the core TGFβ pathway is known, additional components that may further refine signaling remain to be identified. To address this issue, we previously conducted a genetic screen for Sma animals and isolated several new mutants, including *kin-29 *(also known as *sma-11*) [15]. We find that *kin-29 *is able to suppress the long mutant phenotype generated by animals over-expressing the ligand *dbl-1*. Additionally, we observe that *lon-1*, a Sma/Mab pathway target gene whose product shows homology to proteins of the cysteine-rich secretory protein (CRISP) family, is up-regulated in *kin-29(lf) *mutant animals in a similar manner to that seen in a *sma-6 *null mutant background [16,17]. *kin-29 *mutant animals are also developmentally delayed and this defect is partially suppressed by loss of *lon-1 *function. These data suggest that *kin-29 *genetically interacts with Sma/Mab pathway signaling downstream of *dbl-1 *but upstream of *lon-1*.

Several of the Sma/Mab pathway components have been shown to function in the hypodermis to regulate body size morphogenesis. *sma-3*, *sma-6 *and *lon-1*, when specifically expressed in the hypodermis, have been shown to rescue the body size defects associated with each of these loss-of-function mutations [15-18]. The Sma/Mab ligand DBL-1 is primarily expressed in neuronal tissues [8]. It is likely that DBL-1 is secreted from these tissues and targets the hypodermis in order to regulate body size formation. We find that tissue-specific expression of *kin-29(+) *in the hypodermis rescues the small body size phenotype of *kin-29(lf) *animals. In addition, we find that *kin-29(+)*, when expressed in the same tissues as *dbl-1*, also rescues the small body size phenotype of *kin-29(lf) *animals. Therefore, *kin-29 *can function in both hypodermal and neuronal tissues with known Sma/Mab pathway components to regulate body size morphogenesis.

In order to understand how *kin-29 *functions in Sma/Mab pathway signaling, we undertook the molecular characterization of *kin-29*. It is encoded by F58H12.1, which has an N-terminal kinase domain and a novel C-terminal region. Its kinase domain makes it a distant member of the EMK kinase family, which modulates microtubule organization. *kin-29 *has a role in olfaction [19], suggesting that the ability to sense environmental signals influences body size regulation.

## Results

### *kin-29 *encodes a serine threonine kinase

Mutations in members of the Sma/Mab TGFβ-like signaling pathway result in animals that are phenotypically smaller than wild-type. These Sma/Mab mutants are approximately 70% the size of their wild-type counterparts [8,9,11,12,14]. Based on this small body size phenotype, we set out to isolate additional loci which when mutated resulted in small animals. From an F2 EMS screen of N2 wild-type animals, we identified *kin-29(wk61*) [15]. *kin-29(lf) *animals are small, like known Sma/Mab pathway components (Table 1). However, unlike the known pathway components, *kin-29 *does not possess male tail ray fusions or crumpled spicules, suggesting that *kin-29 *is involved in regulation of body size morphogenesis but not male tail development.

**Table 1:** Body size measurements of *kin-29 *alleles

Genotype*	Perimeter (mm)**	%N2	n
N2	2.60 ± 0.14		45
*sma-6(wk7)*	1.85 ± 0.20	71%	46
*kin-29(wk61)*	1.97 ± 0.19	76%	41
*kin-29(oy38)*	2.20 ± 0.18	85%	42
*kin-29(oy39)*	1.98 ± 0.22	76%	38
*lon-1(wk50)*	2.72 ± 0.19	105%	48
*lon-1(wk50);kin-29(wk61)*	2.49 ± 0.17	96%	35

* All animals were measured 48 hours after L4.

** Data are means ± std.

In comparison to N2, all animals are significantly different in size (p < 0.0001).

*kin-29 *was mapped to linkage group X between *unc-2 *and *fax-1*. Appropriate YACs, cosmids, and DNA fragments were used to rescue the gene. The longest cDNA available, y293c7 (Y. Kohara, National Institute of Genetics), spanning this open reading frame was obtained. Based on the ORF sequenced from y293c7, a 10 kb region of genomic DNA containing *kin-29 *was fused in frame with GFP. This construct, *kin-29p::kin-29 gfp*, was then injected into *kin-29 *mutant animals and conferred rescue (Table 3).

**Table 3:** Rescue of *kin-29(wk61) *by promoter fusion constructs

Genotype*	Perimeter (mm)**	%N2	n
*kin-29(wk61)*	2.06 ± 0.13	79%	36
*kin-29(wk61);kin-29p::kin-29:gfp*	2.60 ± 0.12	99%	33
*kin-29(wk61);elt-3p::kin-29:gfp (hypodermal)*	2.41 ± 0.19	92%	35
*kin-29(wk61);dbl-1p::kin-29:gfp (neuronal)*	2.47 ± 0.18	94%	35

* All animals were measured 48 hours after L4.

** Data are means ± std.

In comparison to *kin-29(wk61)*, all animals are significantly different in size from control (p < 0.0001).

**Figure 1:**
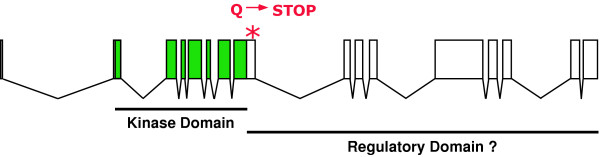
***kin-29 *encodes a serine-threonine kinase. **Schematic of *kin-29 *exon/intron structure including 16 exons. The shaded region at the N-terminus consists of the kinase domain with the Q-to-stop mutation of *kin-29(wk61) *shown. The function of the C-terminus has not yet been determined but it may act as a regulator of kinase activity.

We searched for molecular lesions in *kin-29(wk61)*. Genomic DNA spanning the entire coding region of *kin-29 *was isolated from *kin-29(wk61) *animals, sequenced and compared to sequence obtained from EST y293c7. Sequence analysis reveals *kin-29 *to consist of 16 exons that encode a protein of 822 amino acids in length (Fig. 1). A mutation found in the eighth exon of *kin-29(wk61) *changes a single nucleotide from cytosine to thymine. This change results in a premature termination codon and a truncated protein of 273 amino acids. While this work was in progress, *kin-29 *was cloned as a modifier of olfactory gene expression [19]. Two alleles from that study, *kin-29(oy38) *and *kin-29(oy39)*, result from a 526 bp deletion, which is replaced by sequence found on LG X, and a missense mutation in the kinase domain, respectively [19].

**Figure 2:**
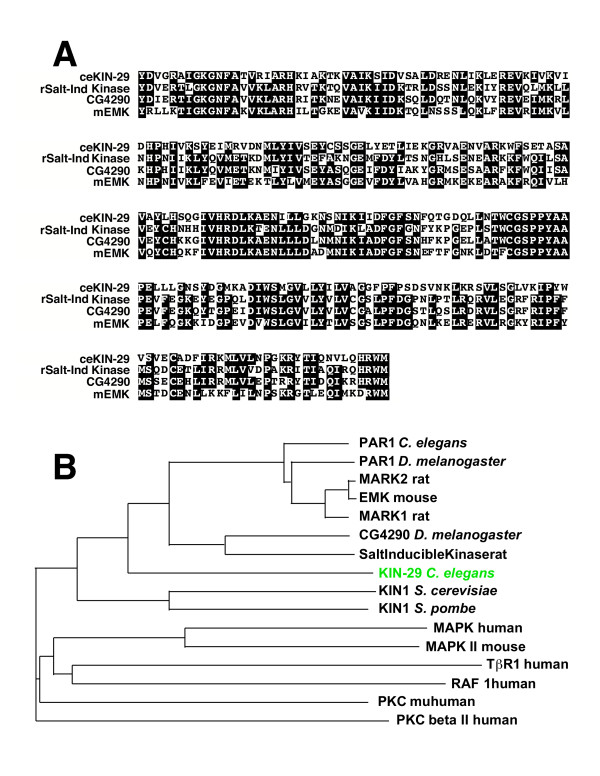
**Molecular analysis of the kinase domain. **(A) Sequence alignment between the kinase domains of KIN-29 (~amino acids 16–267), rat salt-induced kinase (rSalt-Ind Kinase), Drosophila CG4290 (a salt-induced kinase member), and mouse EMK (mEMK). Identical matches in three of the four sequences are indicated by white letters. (B) Dendrogram showing the relationship between the kinase domain of KIN-29 (~amino acids 16–267), and several additional kinases.

*kin-29 *encodes a predicted serine-threonine kinase. Within its kinase domain, KIN-29 is homologous to members of the ELKL motif kinase (EMK) family and salt-induced kinase family (~66% identity)(Fig. 2A,2B). Members of the EMK family include *C. elegans *PAR-1, *Drosophila *PAR-1, *S. pombe *KIN-1, and mammalian MARK (microtubule-affinity-regulating kinase) [20-23]. EMK family members have been shown to affect cell polarity as well as microtubule stability [20,22,23]. The kinase domain of KIN-29 also shows significant homology salt-induced kinases [24,25]. Salt induced kinases (SIK) were cloned from subtractive libraries derived from genes expressed in the adrenal glands after high salt diets in rat. The biological function of these kinases is not clear. The C-terminal domain of KIN-29 is more divergent and shows little homology with domains in other kinases.

### KIN-29 encodes a functional kinase

**Figure 3:**
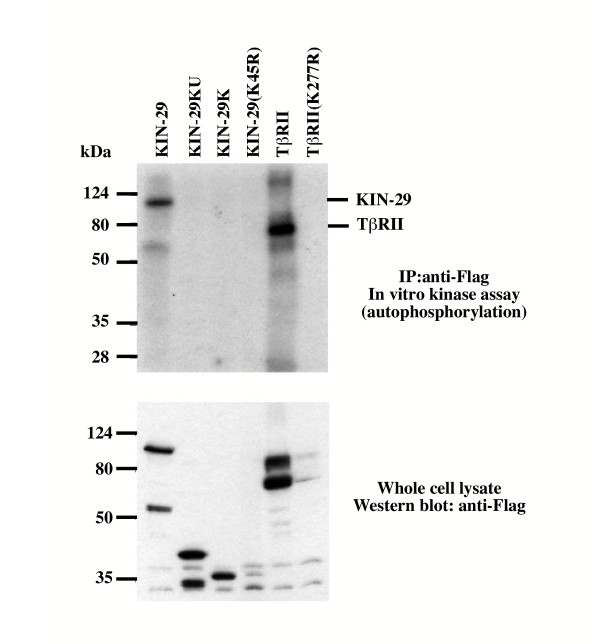
**KIN-29 is a functional kinase. **293T cells were transfected with C-terminal flag tagged *kin-29 *constructs (top panel). KIN-29-KU contains amino acids 1–354, which includes the kinase domain and UBA domain. KIN-29K contains amino acids 1–300 which includes only the kinase domain and KIN-29K(K45R) contains amino acids 1–300 with a point mutation at position 45 that changes a lysine to an arginine. FLAG-tagged proteins were immunoprecipitated using anti-Flag antibody and *in vitro *kinase assays performed. Full length *KIN-29 *is capable of autophosphorylation similar to mammalian Tβ RII (TGFβ type II RSK) (top panel). Truncating the C-terminal domain of *KIN-29 *prevents autophosphorylation.

In order to assess whether KIN-29 acts as a functional kinase, 293T cells were transfected with either C-terminal FLAG-tagged full length *kin-29 *or various constructs, which truncate the carboxy terminal region of the protein. As controls, both the kinase active and kinase inactive mammalian TGFβ type II RSK were also transfected. Lysates were immuniprecipitated with anti-Flag antibody and *in vitro *kinase assays performed. We observe that full length KIN-29 is capable of autophophorylation (Fig. 3). However, when we truncate the C-terminal domain, we find that the kinase domain, along with the ubiquitin-associated domain (UBA) or the kinase domain (with or without lysine 45 changed to arginine) are no longer capable of autophosphorylation. Lysine 45 is a conserved residue essential for catalytic function in kinases. This indicates that the C-terminal domain is required for autophosphorylation. The C-terminal domain could be required for kinase activity or it may simply be the substrate for autophosphorylation. Lanjuin and colleagues have previously shown that animals possessing a mutation (oy39) in the kinase domain have a small body size [19], indicating that the kinase domain is required for proper body size.

### Placement of *kin-29 *in the Sma/Mab pathway

Epistasis between *kin-29 *and *dbl-1 *or *lon-1 *was examined in order to determine the relationship between *kin-29 *and Sma/Mab pathway signaling. Double mutant analysis between several of the known pathway components and *lon-1 *results in animals that are long (Lon) [16,17], making it the most downstream gene in the pathway. We examined *lon-1(wk50); kin-29(wk61) *double mutants to determine whether *kin-29 *can be placed in a similar position in the pathway as the current Sma/Mab components. We find that double mutants are longer than the single mutants of *kin-29(wk61)*, suggesting that *lon-1 *suppresses the Sma phenotype of *kin-29 *(Table 1).

Next, we examined the relationship between *dbl-1 *and *kin-29*. Over-expression of *dbl-1 *results in Lon animals, suggesting more ligand causes an increase in the TGFβ signal output. When *dbl-1 *over-expressing animals are crossed into *sma-2*, *sma-3*, *sma-4*, *sma-6 *or *daf-4 *mutant backgrounds, the Lon phenotype is suppressed [8] and the animals are Sma. This places the type I receptor and the Smads downstream of the ligand, *dbl-1*. When *dbl-1 *is over-expressed in a *kin-29(wk61) *background, the animals are also Sma (Table 2). Additionally, using a weak allele of *sma-6*, we generated a *sma-6(e1482)unc4(e120); kin-29(wk61) *double mutant and examined its body size. We find that these animals are similar in size to that observed for *sma-6(e1482)unc4(e120) *mutants alone suggesting that *sma-6 *and *kin-29 *may not function in parallel pathways (Table 2). This indicates that *kin-29 *behaves in a manner consistent with known Sma/Mab pathway signaling molecules and is likely to function within this signaling cascade.

**Table 2:** *kin-29 *suppresses the *dbl-1 *over-expression phenotype

Genotype*	Perimeter (mm)**	%N2	n
*kin-29(wk61)1, 2)*	1.90 ± 0.15	73%	42
*ctIs40 [pTG96(sur-5::gfp)]dbl-1(+)2*	2.76 ± 0.12	106%	42
*kin-29(wk61); ctIs40 [pTG96(sur-5::gfp)]1*	1.84 ± 0.20	71%	51
*sma-6(e1482) unc-4(e120)3*	1.47 ± 0.13	57%	37
*sma-6(e1482) unc-4(e120);kin-29(wk61)3*	1.48 ± 0.17	57%	38

* All animals were measured 48 hours after L4.

** Data are means ± std.

1-Animals are not significantly different from each other (p > 0.05).

2-Animals are significantly different from each other (p < 0.0001)).

3-Animals are not significantly different from each other (p > 0.05).

**Figure 4:**
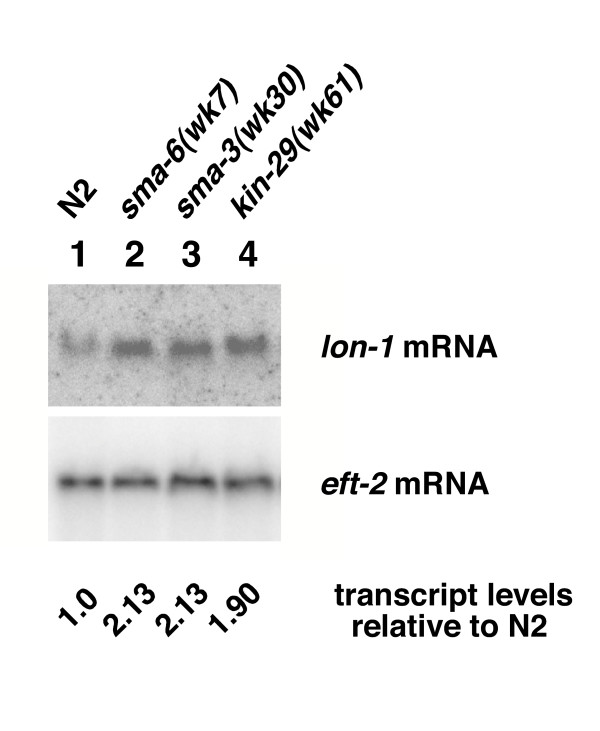
**mRNA levels of the Sma/Mab target gene, *lon-1*, are negatively regulated in *kin-29(wk61) *animals. **Northern blot showing *lon-1 *mRNA expression observed in mixed stage populations of N2, *sma-6(wk7)*, *sma-3(wk30)*, and *kin-29(wk61) *animals. Sma/Mab pathway mutants *sma-6(wk7) *and *sma-3(wk30) *show an up-regulation of the *lon-1 *transcript (lanes 2 and 3). Similarly, *kin-29(wk61) *also shows an increase in *lon-1 *mRNA (lane 4). Elongation factor-2 (*eft-2*) was used to control for amounts of RNA loaded per lane. Levels of mRNA were quantitated using a phosphorimager and IQMacv1.2 software. See Materials and Methods for details on relative transcript level calculations.

Since *lon-1 *is genetically downstream of the Sma/Mab pathway signaling, we examined whether *lon-1 *mRNA levels are altered in *kin-29 *mutants. We have previously shown that *lon-1 *mRNA levels are up-regulated in *sma-6(wk7) *mutants and down-regulated in animals that over-express *dbl-1 *[16,17]. To test whether *lon-1 *mRNA is regulated in *kin-29 *animals, we examined *lon-1 *mRNA levels in a *kin-29(wk61) *background (Fig. 4). *kin-29(wk61) *animals show an increase in the expression level of the *lon-1 *transcript. This increase is comparable to that previously observed in *sma-6(wk7) *mutant animals [16].

*kin-29 *has been shown to affect the expression of a subset of olfactory receptor genes [19]. Several olfactory receptors expressed in AWB, ASH and ASK sensory neurons were either reduced or up-regulated in the *kin-29 *mutant background. Given that *kin-29 *affects mRNA levels of these olfactory receptors, we asked whether *kin-29 *alters the mRNA expression levels of the Sma/Mab components. We examined *sma-6 *mRNA expression (the type I receptor) in a *kin-29 *background and find no changes in expression levels of *sma-6 *mRNA. Because *kin-29 *and *dbl-1 *expression patterns overlap, we examined mRNA expression levels of *dbl-1 *in a *kin-29 *mutant background. We find that *kin-29 *does not affect *dbl-1 *mRNA expression levels (data not shown).

### *kin-29 *expression is diverse and dynamic

**Figure 5:**
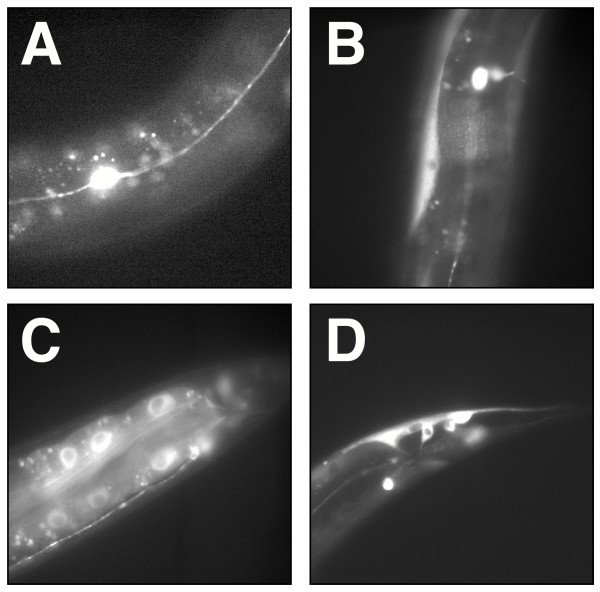
**Expression patterns of *kin-29p:: kin-29:gfp *rescuing construct in wild-type animals. **Animals shown are L4 stage photographed at 63x. *kin-29 *promoter fusion constructs are expressed throughout development: (A) in the CAN neuron, (B) in body wall muscle, (C) in the intestine, and (D) in cells in the tail.

In efforts to elucidate the function of *kin-29 *in TGFβ signaling, we examined its expression pattern. A construct consisting of the *kin-29 *promoter and coding region fused in frame to *gfp *was injected into *kin-29(wk61) *animals. As described above, this construct was able to rescue the small body size phenotype of *kin-29(wk61) *to wild-type (Table 3). Upon examination of the expression pattern, we observe KIN-29 to be localized to various tissue types (Fig. 5). Most notably, KIN-29 is seen in several neuronal cells in the head and tail throughout the course of development. Several of the sensory neurons found in the head express KIN-29, including ASH, AFD and ASI [19]. Additional neuronal staining is observed in both CAN cells and the ventral nerve cord (Fig. 5A). We find expression both in pharyngeal and body wall muscle (Fig. 5B). During the L1, L3 and L4 stages, we see expression throughout the intestine both in the nuclei and to a lesser extent in the cytoplasm (Fig. 5C) and in cells in the tail (Fig. 5D). This intestinal expression is rarely seen in later stages of development. Occasionally, expression is seen in vulval muscles as well.

### Hypodermal and neuronal expression of *kin-29 *rescues the small body size phenotype

In order to determine where *kin-29 *activity is required, we examined the body size of *kin-29(lf) *animals transformed with constructs expressing *kin-29 *in specific tissues. *elt-3 *and *rol-6 *promoters drive expression in the hypodermis, while *dbl-1 *drives expression in a subset of neurons. All three promoters were fused to *kin-29 *genomic DNA sequences. Each of these constructs was injected into *kin-29(wk61) *animals and transgenic strains were analyzed for body size. Several of the Sma/Mab pathway components, *sma-3*, *sma-6*, and *lon-1*, when specifically expressed in hypodermal tissues, rescue the body size defects associated with loss-of-function mutations in each of these genes [16-18,26]. Using the *rol-6 *and *elt-3 *promoters to drive hypodermal expression of the genomic region of *kin-29 *results in rescue of the small body size phenotype of *kin-29(wk61*). Since KIN-29 expression overlaps that of the Sma/Mab pathway ligand DBL-1 in the amphid neurons, ventral nerve cord, CAN cells and body wall muscle, we reasoned that KIN-29 and DBL-1 may function together in the same tissues to regulate body size morphogenesis [8,9]. We find that *kin-29 *under the control of the *dbl-1 *promoter rescues the small body size phenotype of *kin-29(wk61) *animals (Table 3). These data suggest that KIN-29 functions in neuronal and hypodermal tissues to regulate body size morphogenesis (Table 3 and data not shown).

### *kin-29 *mutants are small, have delayed growth rates, and reduced brood sizes

**Figure 6:**
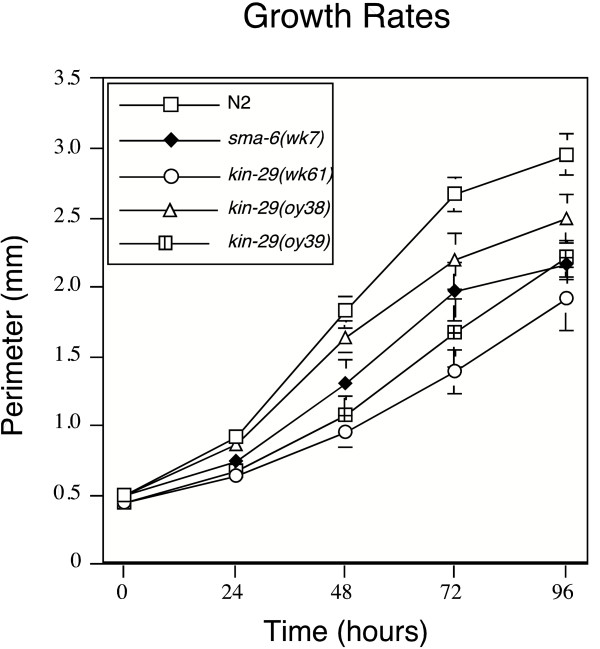
**The small body size phenotype of *kin-29*animals is a result of defects in postembryonic development. **N2, *sma-6(wk7)*, *kin-29(wk61)*, *kin-29(oy38) *and *kin-29(oy39) *were hatched and synchronized as L1 animals. L1 animals were measured at time zero and then at 24-hour time points spanning a 96 hour period. *kin-29 *animals are developmentally delayed and over time, *kin-29(lf) *animals never reach a wild-type body size. Perimeter measurements for at least 22 animals were averaged at each time point. Error bars represent standard deviation values. Values for N2 and *kin-29 *mutants are significantly different (p < 0.001).

The growth properties of Sma/Mab animals differ from other small animals. For example, mutants of the spectrin gene, *sma-1*, which have been shown to affect embryonic elongation but not thought to be involved in TGFβ signaling, are approximately 50% the size of wild-type animals at hatching [27]. This is in contrast to the body size of L1 animals mutant in known Sma/Mab pathway components. For example, *sma-3*, *sma-6*, *dbl-1*, and *lon-1 *are indistinguishable in length from wild-type L1 animals at hatching [26]. This suggests that the Sma/Mab pathway components are defective in post-embryonic rather than embryonic stages of development. Sma/Mab pathway mutant animals grow at a slower rate as development progresses through the later larval stages into adulthood [26]. There is no defining switch during development that regulates body growth. We tested whether *kin-29 *mutations cause body size defects in a similar manner to Sma/Mab pathway mutations or whether *kin-29 *possessed embryonic defects. We examined the body size of *kin-29 *mutant animals in comparison to *sma-6(wk7) *and N2 animals at hatching and then at 24 hour intervals to 96 hours. We find that all three alleles of *kin-29 *are similar in length at the L1 stage to N2 animals. This is also what we observe for *sma-6(wk7) *which suggests that *kin-29 *delays growth post-embryonically, as do Sma/Mab pathway components (Fig. 6). The Sma body size of *kin-29 *is therefore due to a delay in development in later larval stages.

In addition, we find that *kin-29 *grows more slowly than N2 and Sma/Mab pathway mutants do. Animals hatched and grown at 20°C were scored based on their developmental stage after 72 hours. We find that 99% of wild-type animals are adults at this time point, while only 2% of *kin-29(wk61) *animals are adults (Table 4). Lanjuin and colleagues report a similar observation; 98% of wild-type animals hatched and grown at 25°C for 3 days were adults in comparison to approximately 24% of *kin-29(oy38) *animals [19]. We asked if *lon-1(lf) *could suppress the developmental delay characteristic of *kin-29(wk61) *animals (Table 4). *lon-1(wk50) *mutants on their own show a slight delay in development, but which is distinguishable from the Sma/Mab mutants. In the double mutant *lon-1(wk50);kin-29(wk61)*, we find that the developmental defect of *kin-29(wk61) *can be partially suppressed by *lon-1(wk50)*. This result is consistent with our conclusion that *lon-1 *is genetically downstream of *kin-29*.

**Table 4:** *lon-1 *partially suppresses the developmental defect of *kin-29(wk61)*

	% Adult animals	% Adults 4 animals
	
Genotype	20°C	20°C
N2	99 (185)	99 (185)
*lon-1(wk50)*	64 (245)	80 (245)
*kin-29(wk61)*	2 (475)	43 (475)
*lon-1(wk50);kin-29(wk61)*	40 (202)	63 (202)

Number of animals scored is shown in parentheses.

We observed that Sma/Mab pathway mutants have a reduced brood size. In addition to the developmental defects, *kin-29(wk61) *also has a reduced brood size (Table 5). Like *sma-6(lf) *and *lon-1(lf)*, *kin-29(wk61) *shows a brood size approximately 30% the size of that seen in wild-type animals. We find that *sma-6(wk7) *and *lon-1(wk50) *along with *kin-29(oy38) *and *kin-29(oy39) *have a reduction in brood size as well. Although brood size is affected, embryonic survival rate appears to be normal.

**Table 5:** Brood size analysis of *kin-29 *alleles

Genotype	% of wild-type brood size
N2	100 (270)
*sma-6(wk7)*	64 (172)
*lon-1(wk50)*	81 (219)
*kin-29(wk61)*	32 (86)
*kin-29(oy38)*	81 (218)
*kin-29(oy39)*	80 (217)

Number of eggs scored for each genotype is shown in parentheses.

### *kin-29 *affects dauer pathway signaling

Several components of the Sma/Mab pathway have been shown to genetically interact with members of the dauer pathway [9,14]. The dauer-constitutive (Daf-c) phenotype of the type I receptor *daf-1 *is enhanced by mutations in *sma-6*. At 15°C, *daf-1 *mutant strains exhibit a very weak dauer-constitutive phenotype. However, *sma-6(wk7); daf-1(m40) *mutants show a 50% increase in the number of dauered animals at 15°C [14]. In addition, double mutants between the ligand *daf-7(e1372) *and either *dbl-1(kk3) *or *sma-2(e502) *also have been shown to enhance the weak Daf-c phenotype of *daf-7(e1372) *at 20°C [9]. These data suggest that there is some crosstalk between the Sma/Mab pathway and the TGFβ-like *daf-7 *dauer pathway.

**Figure 7:**
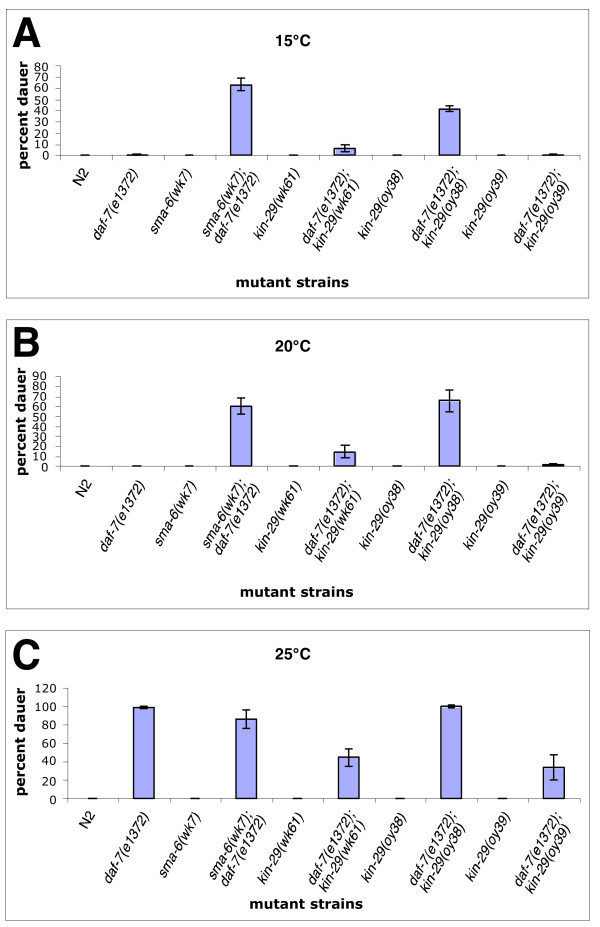
**Interaction between *kin-29 *and the *daf-7 *TGFβ-like pathway. **N2 or dauered animals are not seen at 15°C or 20°C (A, B). *daf-7(e1372) *mutant animals form constitutive dauers at 25°C (C). *kin-29 *mutants can both suppress and enhance the dauer constitutive phenotype of *daf-7(e1372)*. At 25°C, *daf-7(e1372); kin-29(wk61) *and *daf-7(e1372); kin-29(oy39) *mutants show suppression of the Daf-c phenotype while *daf-7(e1372); kin-29(oy38) *mutants do not (C). At 15°C and 20°C, *daf-7(e1372); kin-29(oy38) *mutants show an enhancement in dauer formation similar to that observed in *sma-6(wk7); daf-7(e1372) *animals(A, B). Additionally, *daf-7(e1372); kin-29(wk61) *mutants show a mild enhancement of the Daf-c *daf-7 *phenotype (A, B).

Based on these findings, we examined the effects of the *kin-29 *alleles on dauer formation. Double homozygotes were made between *daf-7(e1372) *and each of the three alleles of *kin-29*. Genetic interactions were analyzed at 15°C, 20°C and 25°C. For comparison, *daf-7(e1372) *mutants raised at 25°C show almost 100% dauered animals compared to no dauered animals at 15°C or 20°C. At 15°C and 20°C, *kin-29(oy38) *is able to enhance dauer formation of *daf-7(e1372) *similar to the enhancement observed in *sma-6(wk7); daf-7(e1372) *mutant animals (Fig. 7A,B). We also see that *kin-29(wk61) *shows a weak enhancement of dauer formation while the missense mutant *kin-29(oy39) *shows no genetic interaction at all. These results are consistent with genetic interactions previously observed between Sma/Mab and dauer pathway components. However, at 25°C, we find that *kin-29 *can also suppress the constitutive dauer formation of *daf-7(e1372)*. *kin-29(wk61) *and *kin-29(oy39) *are able to suppress the Daf-c defects of *daf-7(e1372) *while *kin-29(oy38) *does not (Fig. 7C).

## Discussion

### KIN-29 functions in hypodermal and neuronal tissues to regulate body size

Expression of the Sma/Mab pathway components in the hypodermis is sufficient to rescue the body size defects seen in mutants. Specific expression of *sma-3*, *sma-6 *and *lon-1 *in the hypodermal tissues has been shown to restore body length in these respective mutant animals [16-18,26]. This implies that body size is regulated largely via hypodermal function. Our work presented here further supports that *C. elegans *body size is regulated in hypodermal tissues. When the genomic region of *kin-29 *is specifically expressed in the hypodermis, under the control of the *elt-3 *and *rol-6 *promoters, we see that the small body size phenotype of *kin-29(wk61) *is partially rescued. Although KIN-29 functions in the hypodermis to regulate body size morphogenesis, we do not see KIN-29::GFP, under the control of its own promoter, expressed in the hypodermal tissues, suggesting that KIN-29 expression levels are relatively low in these tissues.

DBL-1 is expressed primarily in neurons, which includes several amphid and pharyngeal neurons, ventral nerve cord, and CAN cells [8,17]. Since KIN-29 expression closely parallels that seen for DBL-1, we also examined whether the *dbl-1 *promoter driving *kin-29 *genomic sequences is capable of rescuing the body size defect of *kin-29(wk61)*. When *kin-29 *is expressed in the same tissues as *dbl-1*, we observe partial rescue of the small body size defect seen in *kin-29(lf) *animals. It has been demonstrated that *kin-29 *under the control of the *unc-14 *and *odr-4 *promoters is able to rescue the body size defect of *kin-29 *mutant animals [19]. *unc-14 *is expressed in all neuronal cells while *odr-4 *is expressed in a subset of the sensory neurons including the AFD neurons where DBL-1 and KIN-29 are also expressed [28,29]. This suggests that neuronal expression of KIN-29 is also sufficient to regulate body size morphogenesis. Determining how this occurs will require further study.

### *kin-29 *is a tissue specific factor that affects the Sma/Mab pathway

Mutations in the ligand *dbl-1*, the receptors *sma-6 *and *daf-4*, and Smads *sma-2*, *sma-3 *and *sma-4*, result in animals that are approximately 70% the size of wild-type animals [8,9,11,12,14]. Additional defects are seen as male tail ray fusions and crumpled spicules. However, the negatively regulated Sma/Mab target gene *lon-1*, suppresses the small body size phenotype of *sma-2*, *sma-3, sma-4 *and *sma-6 *but not the male tail defects observed in each of these loss-of-function mutants [16,17]. This implies that Sma/Mab pathway signaling may branch downstream of the Smads to regulate a subset of genes that control body size morphogenesis while others specifically affect male tail development. We find that *kin-29(lf) *animals do not posses ray fusions or crumpled spicules and may exert its effects upstream of this branch point in the signaling pathway. In addition, we see that *lon-1 *is genetically downstream of *kin-29 *and that *kin-29 *suppresses the Lon phenotype associated with over expression of *dbl-1*. Taken together, this data suggests that *kin-29 *may function in tissues with Sma/Mab pathway components to regulate body size but not male tail formation.

### The EMK kinase family and *kin-29*

Members of the EMK family have been shown to affect cell polarity and microtubule stability. Mammalian MARK phosphorylates microtubule associated proteins and has been shown to destabilize microtubules when over expressed in CHO cells [23]. *Drosophila *PAR-1 influences the cytoskeletal organization of the oocyte [21]. In wild-type *Drosophila *oocytes, the microtubules are arranged in an anterior to posterior gradient with no microtubules observed at the most posterior region of the oocyte. Microtubules in *Drosophila par-1 *mutants, however, are organized around the cortex of the oocyte. In this reorganization, microtubules are now observed in the most posterior region of the oocyte. In addition, posterior localization of *Drosophila oskar *is dependent on microtubule polarity. *oskar *is mislocalized in *dpar-1 *mutants, further supporting the involvement of *dpar-1 *in regulating microtubule dynamics. *C. elegans *PAR-1 regulates the early asymmetrical cell divisions of the embryo but has not been shown to have any affects on the microtubule network [20]. KIN-29 only shows homology to the EMK family members within its N-terminal kinase domain, indicating that KIN-29 is a more distantly related member of the EMK family. However, lack of homology between the C-termini of KIN-29 and EMK family proteins suggests that KIN-29 activity may diverge from that observed for members of this family.

### How does *kin-29 *function?

We have observed that KIN-29 functions in both neuronal and hypodermal tissues. How *kin-29 *functions in each of these tissues to regulate body size morphogenesis is unclear. Since *kin-29 *encodes a kinase it might act to regulate the activities of a variety of molecules that affect Sma/Mab pathway signaling. Recently, it has been shown that several olfactory receptors are misexpressed in *kin-29(lf) *animals, suggesting that KIN-29 may regulate proper expression levels of various genes [19]. One model is that KIN-29 phosphorylates a transcription factor and/or co-factor, which leads to the transcriptional mis-regulation of some component important for Sma/Mab pathway signaling. We have examined the expression levels of the Sma/Mab ligand *dbl-1 *and the type I RSK *sma-6 *and do not see any alteration in their levels of expression. However, this does not rule out that other genes that impinge on pathway signaling might be affected at the transcriptional level in neurons and hypodermal cells. Alternatively, KIN-29 may function in microtubule dynamics as described above [21,23]. *kin-29 *might therefore influence microtubule (MT) organization in both neuronal and hypodermal tissues and affect Sma/Mab pathway signaling in each of these cell types.

### Dauer interactions show that *kin-29(lf) *mutants may not sense external cues properly

We have shown that KIN-29 helps to promote dauer formation at 25°C and to suppress dauer formation at 15°C. *ttx-3*, a LIM homeobox gene, shows a similar genetic interaction with *daf-7 *[30]. Like *kin-29*, *ttx-3 *single mutants do not affect dauer formation. *daf-7(e1372); ttx-3(ks5) *double mutant animals show an enhanced Daf-c phenotype of *daf-7 *at 15°C while suppressing it at 25°C. In wild-type animals, high temperatures contribute to dauer formation, while lower temperatures suppress dauer formation. *ttx-3 *decouples both hot and cold inputs from the dauer pathway, and *kin-29 *may act similarly [30]. *tph-1*, a tryptophan hydroxylase involved in the synthesis of serotonin, has been shown to form 10–15 % dauers in the presence of food and this defect is not dependent on temperature which implicates serotonergic signaling in modulating temperature sensitive dauer arrest [31]. *tph-1 *is also able to enhance the constitutive dauer phenotype of *daf-7 *mutants at 15°C similar to the enhancement observed for *kin-29 *[31]. Sze and colleagues did not examine the affects of *tph-1 *mutants on *daf-7 *at 25°C. They did show, however, that over-expression of *tph-1 *in a *daf-7 *background at 25°C suppresses the Daf-c phenotype of *daf-7*. This is opposite to what we observe for *kin-29*. Although there are some similarities between *tph-1 *and *kin-29*, *tph-1(mg280); kin-29(oy38) *double mutants are synthetic-*daf *at 20°C and 25°C suggesting that *kin-29 *may not function in a linear pathway with *tph-1 *but rather parallel to *tph-1 *[19]. *tph-1 *has also been shown to regulate the expression of *daf-7 *while *kin-29 *does not, suggesting that *kin-29 *functions downstream or in parallel to *daf-7 *production to affect TGFβ signaling outputs [19,31]. *kin-29 *may partly influence dauer formation through a serotonin mediated pathway and a non-serotonin mediated pathway such as the Sma/Mab pathway. The Sma/Mab pathway has been shown to influence dauer formation in combination with the TGFβ-like *daf-7 *pathway [9,14].

Starved animals are smaller than animals grown with abundant food supplies, indicating that environmental conditions influence body size morphogenesis [13]. *Drosophila *S6 kinase mutants are smaller in body size due to decreased cell size, which is similar to the body size defect observed in nutrient deprived flies [32,33]. It is thought that S6 kinase alters cell growth in response to nutrients and growth factors by regulating the efficiency of the translational apparatus [34]. Recently, it has been shown that a deletion found within the *C. elegans *homolog of S6 kinase *(sv31) *also results in a reduced body size in the adult stage (J. Friberg and S, Tuck, personal communication). S6 kinase *(sv31) *and *kin-29(lf) *animals also show other similar phenotypes, including reduced brood size and slow growth defects. In addition, fat accumulation is also observed in S6 kinase *(sv31) *animals similar to that observed in dauered animals. Previously, it has been demonstrated that animals that show pheromone hypersensitivity are unable to sense food or temperature signals properly [35-39]. Recently, *kin-29 *mutants have been shown to be hypersensitive to pheromone [19]. In addition, *kin-29 *mutants also possess hyperforaging activity in the presence of abundant food supplies [19]. Hyperforaging is normally observed in animals that have been deprived of food. These defects suggest that *kin-29(lf) *mutant animals may not sense food or temperature signals properly and this may influence body size regulation. Taken together, this evidence supports an environmental role in regulation of body size. *kin-29 *may function to transmit these environmental cues to the Sma/Mab TGFβ signaling pathway, thereby affecting proper body size morphogenesis.

## Conclusion

In this study, *kin-29 *was identified in a genetic screen designed to identify modifiers of body size in *C. elegans*. Mutants in *kin-29 *result in small animals, and we show that *kin-29 *affects the *dbl-1 *signaling pathway in *C. elegans*. *kin-29 *also modifies phenotypes from a second TGFβ pathway in *C. elegans*, the dauer pathway. Further, we show that KIN-29 does contain kinase activity, and that it is capable of phosphorylating itself. KIN-29 functions in neurons and in the hypodermis to control aspects of body size.

## Methods

### Strains

*C. elegans *strains were grown using standard methods [40]. *kin-29(wk61) *was used for body size rescue experiments. N2, *sma-6(wk7)*, *kin-29(wk61)*, *kin-29(oy38) *and *kin-29(oy39) *were used in generating growth curves [19]. Interactions between *kin-29 *and the dauer pathway were examined using *daf-7(e1372)*. Total RNA used for northern blot analysis was isolated from N2, *sma-6(wk7)*, *sma-3(wk30)*, and *kin-29(wk61*) animals. Epistasis was determined using *kin-29(wk61)*, *lon-1(wk50)*, *dbl-1 *over expressing strain *ctIs40 [pTG96 (sur-5::gfp)]*, and *lon-1(wk50); kin-29(wk61) *mutant animals.

### Isolation of *kin-29(wk61)*

*kin-29(wk61) *was generated from an EMS F2 screen designed to isolate small body size mutants [15]. *kin-29(oy38) *and *kin-29(oy39) *were obtained from P. Sengupta [19].

### Cloning of *kin-29*

*kin-29(wk61) *was mapped to a small region on the X chromosome between *unc-2 *and *fax-1 *using genetic markers and deficiencies. The YAC clone Y76F7 from this interval was purified from total yeast DNA using pulse field gel electrophoresis. Injection of YAC Y76F7 into *kin-29(wk61) *rescued the small body size phenotype. Next DNA from cosmids contained within the region of Y76F7 was isolated and transgenic lines were generated. Cosmid F58H12, with one predicted open reading frame (ORF), conferred rescue. ESTs spanning this region were obtained from the *C. elegans *cDNA project (Y. Kohara, National Institute of Genetics). The longest EST, y293c7, was sequenced and shows minor differences from the Genefinder prediction. A 10 kb genomic region fused in frame to GFP, which contained the corresponding sequence from y293c7, was generated by PCR as described below. This *kin-29p::kin-29:gfp *fusion construct rescued the small body size of *kin-29(wk61)*. Genomic DNA from homozygous *kin-29(wk61) *animals was sequenced. Two independent PCR amplifications were generated for each of the three regions spanning the *kin-29 *coding region using the following primer sets: CGCTGCGGCCGCTTCAGGCGCCGCCACACCAA/ CGCCGCTGCAGCCGCCGGCAACGAGAATGTA; CGCTGCGGCCGCCCAAGCCAACGTTGCAGGTA/ CGCCGCTGCAGGATAACATGCTCCACTGGCTA; CGCTGCGGCCGCCACCGCACGGGCTAGATATT/ CGCCGCTGCAGCCATTCACTCCGAGCTCCAG. Each PCR product was digested with Not I and Pst I, subcloned into pBluescript SK+, and sequenced.

### GFP fusion and tissue-specific expression constructs

*kin-29p::kin-29 gfp *contains the 10 kb genomic region of *kin-29 *fused in frame to *gfp*. This construct was generated using the primers CGCGCTGCAGCAGACCATGGACGT GTTTTAATG and CCGGGGATCCTCCGAGCTCCAGCTTGGATCA, digesting with Pst I and BamH I, and inserting the PCR product into the promoterless vector pPD95.75. *kin-29p::gfp *was generated by cloning 1.4 kb upstream of the predicted *kin-29 *ATG into the Hind III and Xba I sites of the GFP insertion vector pPD95.69 (A. Fire, Stanford University). The 1.4 kb piece was generated by PCR using primers CCGGAAGCTTCAGACCATGGACGTGTTTTAATG and CGCGTCTAGATGCAGTGTTGGTGTGGCGGC. Fluorescent GFP expression patterns were examined in larval and adult animals using a Zeiss compound microscope.

*kin-29 *genomic DNA was ectopically expressed in specific tissues using the promoters *rol-6*, *elt-3 *and *dbl-1 *[8,41,42]. The *rol-6 *and *elt-3 *promoters express in the hypodermis, while the *dbl-1 *promoter expresses primarily in neuronal tissues. PCR fragments containing the *elt-3*, *rol-6 *and *dbl-1 *promoters were generated using the primer sets CCGGAAGCTTGTGACACGTTGTTTCACGGTCAT/ CCGGCTGCAGGAAGTTTGAAATACCAGGTAGCCGA, CCGGCTGCAGCTTCGTATTAGATCTCAGCAGC/ CGCGCGTCGACAGTTAGATCTAAAGATATATCCAG, and CCGGCTGCAGCCCGGAAATCACGACCAAATGGGTC/ CGCGCGTCGACAGTTGAGTTGGGCGCATCAGGCAG respectively. *elt-3 *PCR products were digested with Hind III and Pst I while *rol-6 *and *dbl-1 *products were digested with Pst I and Sal I. 7.7 kb PCR fragments comprising *kin-29 *genomic DNA were generated using the primer sets CCCGGGTCGACATGGCTGCGCCACGGCGGCGTAT/CCGGGGATCCTC CGAGCTCCAGCTTGGATCA and CGCGCTGCAGCAGACCATGGACGTGTT TTAATG/ CCGGGGATCCTCCGAGCTCCAGCTTGGATCA and digested with either Sal I and Bam HI or Pst I and Bam HI respectively. Fragments were inserted in frame into the promoterless *gfp *insertion vector pPD95.75. All constructs were injected into *kin-29(wk61) *and transformants were analyzed for body size rescue.

### Analysis of body size, brood size and growth rates

For body size measurements, animals were photographed 48 hours after the L4 stage using a Nikon SMZ-U dissecting microscope set at 3.5× magnification and software from Strata Video Shop (Strata Inc.). Screen dimensions were 680 X 460 pixels. Perimeter analysis was done using Image Pro Plus (Mediacybernetics).

For brood size analyses, single L4 animals were picked to individual plates. Every 12 hours, animals were transferred to new plates to continue egg laying. All eggs were counted. 24 hours later, hatchlings were scored.

For growth rate analyses, animals were synchronized. Gravid animals were treated with a hypochlorite/NaOH solution in order to isolate eggs. The eggs were allowed to hatch in M9 for at least 24 hours. Approximately 30 L1 animals were placed onto plates seeded with OP50. Animals were initially measured at the L1 stage (time zero) and then at 24 hour intervals thereafter. The final time point was taken 96 hours after the L1 stage. Images were obtained and perimeter analyses were performed as described above.

### Genetic interactions with *daf-7(e1372)*

Double mutants were generated between *daf-7(e1372) *and the following mutants: *sma-6(wk7)*, *kin-29(wk61)*, *kin-29(oy38)*, and *kin-29(oy39)*. Gravid animals were placed onto plates well seeded with OP50 and allowed to lay eggs at room temperature. Animals were removed from plates after approximately 30 – 50 eggs were laid. Eggs were allowed to hatch at 15°C, 20°C and 25°C. The number of dauered animals was counted and graphed.

### Northern blot analysis

Total RNA from L4 animals was isolated from N2, *sma-6(wk7)*, *sma-3(wk30) *and *kin-29(wk61) *as described previously (previously described in [16]. Equal amounts (20–30 μg) of total RNA were loaded per lane onto a 1.2% agarose/6.6% formaldehyde gel and resolved by electrophoresis. Samples were transferred to nitrocellulose (Osmonics Inc.) and baked at 80°C for 2 hours. The *lon-1 *and *dbl-1 *probes were generated by digesting both the *lon-1 *cDNA B1.11 and the *dbl-1 *cDNA with Eco RI. The *sma-6 *probe was generated by PCR using the primer set: GCCGCCTCGAGATGAACATCACCTTTATATTTATTCTC/ GCCGCGGATCCTTAAGATTGATTGGTGGCTGAC. Elongation factor-2 and α-tubulin were used as controls to indicate the amount of total RNA loaded per lane. Before probes were added, the nitrocellulose blots were prehybridized with 1 mM EDTA, 0.5 M NaPO4, pH7.2, 7% SDS, and 1% BSA fraction V (Sigma) for at least 30 minutes. Probes were labeled using the Prime It II kit (Stratagene), added to the prehybridization solution, and incubated overnight at 65°C. Blots were washed (1 mM Na_2_EDTA, 40 mM NaPO_4_, pH 7.2, and 1% SDS) at least three times at 65°C for 15 minutes. Each blot was placed onto a phosphorimager screen for at least 48 hours and analyzed using a Molecular Dynamics Phosphorimager (Amersham Pharmacia Biotech) and IQMacv1.2 software. For each band, intensity levels were corrected for background and normalized according to the loading control (*eft-2 *mRNA). Relative transcript levels of the mutants were normalized to the intensity ratio of *lon-1*/*eft-2 *of N2.

### Kinase assays

C-terminally tagged Flag *kin-29 *constructs were generated in the mammalian vector pRK5. KIN-29-KU contains amino acids 1–354 which includes the kinase domain and UBA domain. KIN-29K contains amino acids 1–300 which includes only the kinase domain and KIN-29K(K45R) contains amino acids 1–300 with a point mutation at position 45 that changes a lysine to an arginine. Cell transfection, immunoprecipitation and kinase assays were carried out as previously described [41]. Human 293T cells at 30% confluency were transfected with each construct (2 μg in 100 mm plates) using LipofectAMINE (Life Technologies, Inc.). Forty eight h after transfection, cells were lysed in the lysis buffer (25 mM Tris-HCl, 300 mM NaCl, and 1% Triton X-100). Lysates were immunoprecipitated using anti-Flag antibody M2 (Sigma), washed 3 times in the same buffer and a final wash in the kinase buffer (10 mM HEPES-KOH, pH 7.5, 5 mM MgCl_2_, and 5 mM CaCl_2_). For *in vitro *kinase assays, the immunoprecipitated protein samples were divided into two aliquots. One aliquot was analyzed by anti-Flag western blotting. The second aliquot was subjected to a kinase autophosphorylation assay at room temperature for 30 min in the kinase buffer containing 5 μCi of ^32^P-ATP (5000 μCi/mmol). The reaction was terminated by adding an equal volume of 2 × SDS sample buffer (80 mM Tris, pH 6.8, 3.2% SDS, 16% glycerol, 200 mM dithiothreitol, 0.02% bromphenol blue), then subjected to SDS-PAGE and visualized by autoradiography.

## Authors' contributions

L.L.M. performed the majority of experiments, including the mapping and molecular cloning of *kin-29*. S.C initiated the early phases of mapping *kin-29*. A.F.R. analyzed brood size. C.M.Z. and A.F.R. assisted with generating transgenic nematode lines. H.W. and L.C. provided assistance in construct design and scoring of genetic experiments. X.L. and X-H.F. performed the kinase assays. R. W. P. implemented and supervised the project and R.W.P. and L.L.M. prepared the manuscript.
